# Autoinhibition in Ras effectors Raf, PI3Kα, and RASSF5: a comprehensive review underscoring the challenges in pharmacological intervention

**DOI:** 10.1007/s12551-018-0461-0

**Published:** 2018-09-29

**Authors:** Ruth Nussinov, Mingzhen Zhang, Chung-Jung Tsai, Tsung-Jen Liao, David Fushman, Hyunbum Jang

**Affiliations:** 1 Cancer and Inflammation Program, Leidos Biomedical Research, Inc., Frederick National Laboratory for Cancer Research, National Cancer Institute at Frederick, 1050 Boyles St., Frederick, MD 21702 USA; 2 Department of Human Molecular Genetics and Biochemistry, Sackler School of Medicine, Tel Aviv University, 69978 Tel Aviv, Israel; 3 Biophysics Program, Institute for Physical Science and Technology, University of Maryland, College Park, MD 20742 USA; 4 Department of Chemistry and Biochemistry, Center for Biomolecular Structure and Organization, University of Maryland, College Park, MD 20742 USA

**Keywords:** KRAS, HRAS, NRAS, K-RAS4A, K-RAS4B, Drug, Inhibitor, Allosteric, Activation

## Abstract

Autoinhibition is an effective mechanism that guards proteins against spurious activation. Despite its ubiquity, the distinct organizations of the autoinhibited states and their release mechanisms differ. Signaling is most responsive to the cell environment only if a small shift in the equilibrium is required to switch the system from an inactive (occluded) to an active (exposed) state. Ras signaling follows this paradigm. This underscores the challenge in pharmacological intervention to exploit and enhance autoinhibited states. Here, we review autoinhibition and release mechanisms at the membrane focusing on three representative Ras effectors, Raf protein kinase, PI3Kα lipid kinase, and NORE1A (RASSF5) tumor suppressor, and point to the ramifications to drug discovery. We further touch on Ras upstream and downstream signaling, Ras activation, and the Ras superfamily in this light, altogether providing a broad outlook of the principles and complexities of autoinhibition.

## Introduction

Ras effectors play major roles in cell fate (Ahearn et al. 2011; Barnoud et al. 2017; Donninger et al. 2016; Iwasa et al. 2018; Sanchez-Sanz et al. 2016). Their affinities to Ras, signaling timescales, functions, and mechanisms vary (Herrmann et al. 1996; Jarvis 2011; Karasarides et al. 2001; Liao et al. 2016; Linnemann et al. 2002; Nussinov et al. 2016b; Nussinov et al. 2018c; Owonikoko and Khuri 2013; Pons-Tostivint et al. 2017; Rusanescu et al. 2001; Stephens et al. 2005; Vadas et al. 2011). Current observations suggest that all are autoinhibited at the membrane.

Below, we lay out the principles of autoinhibition and its release, focusing on Raf protein kinase, phosphatidylinositide-3-kinase α (PI3Kα) lipid kinase, and Ras association domain family 5 (RASSF5, a.k.a. NORE1A) tumor suppressor; however, our discussion is general. Cell signaling needs to be sensitive to the environment, and sensitivity is feasible *only* if the difference in the stabilities between the OFF and ON states is small, requiring only a small-scale shift in the equilibrium. The interactions between the target protein and its autoinhibiting segment, domain, or subunit display a continuum; if they are less stable, crystallography (or NMR) is unlikely to capture the autoinhibited conformation; if they are slightly more stable, it will (Huang et al. 2007). This principle clarifies autoinhibition and its release, and underscores the challenge facing pharmacology aiming to sustain autoinhibited states.

**Fig. 1:**
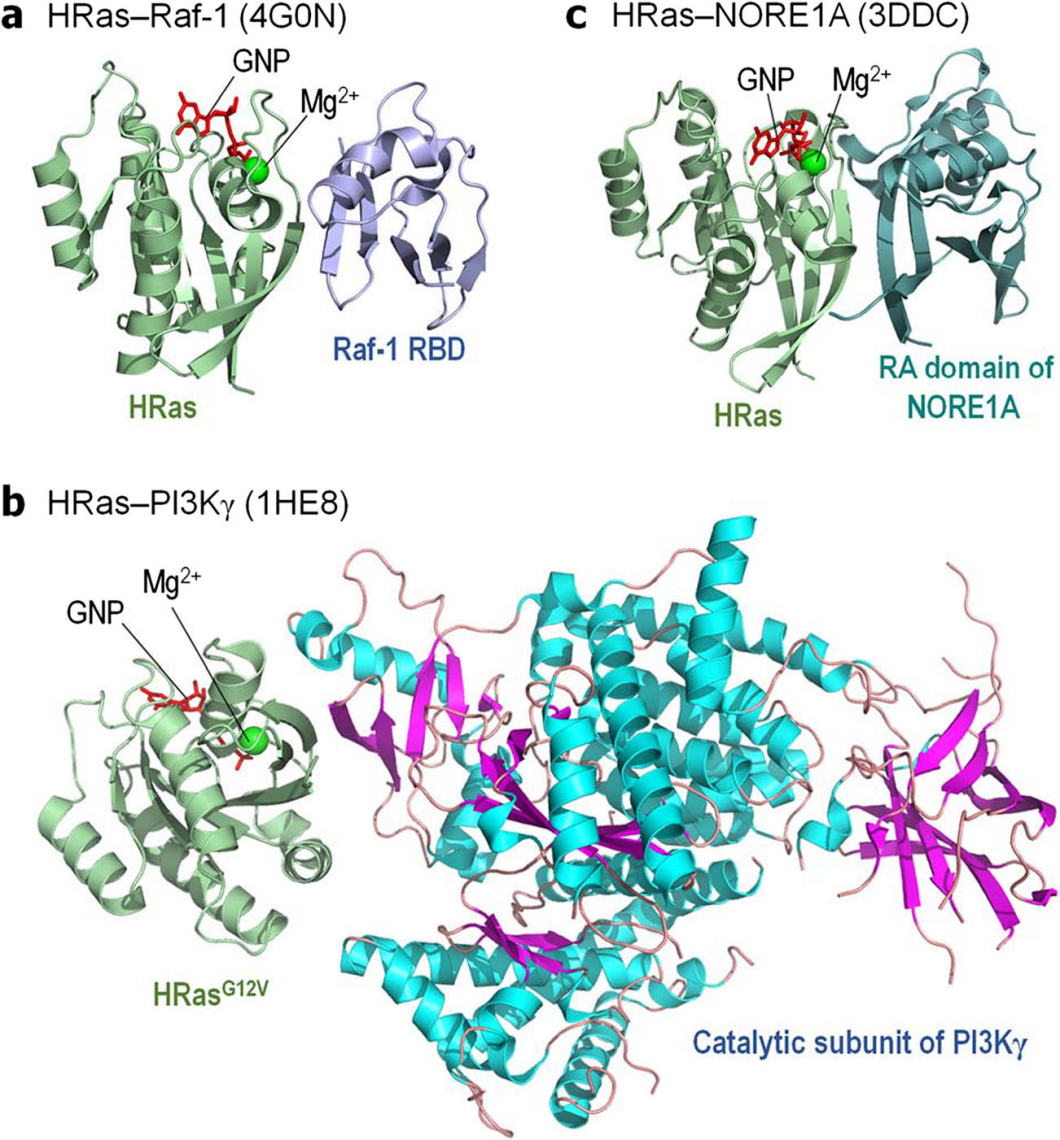
Ras and its effectors. Cartoon representation of the crystal structures of **a** GppNHp-bound HRas interacting with the Ras binding domain (RBD) of Raf-1 (PBD code: 4G0N), **b** GppNHp-bound HRas^G12V^ mutant interacting with the catalytic subunit of PI3Kγ (PBD code: 1HE8), and **c** GppNHp-bound HRas interacting with the Ras association (RA) domain of murine NORE1A (PBD code: 3DDC)

We selected Raf, PI3Kα, and NORE1A as representative Ras targets (Fig. 1) that have some mechanistic structural data and discuss their autoinhibition and its release to the extent that the data permit. Whereas even without the involvement of Ras, the release of the autoinhibition with subsequent kinase domain dimerization are sufficient for full activation of a Raf molecule, and active Ras increases the otherwise minor population of the active species, this is not the case for PI3Kα. In PI3Kα, release of the autoinhibition and Ras binding are two independent, additive components of full activation (Karasarides et al. 2001). To increase the population of the active Raf species, at least two elements are required: (i) spatial proximity of Ras molecules, via Ras nanoclustering (or dimerization/oligomerization) and (ii) high affinity to Ras. Neither is required for PI3Kα, where the release of the autoinhibition and the consequent increase in the population of the active species is via high affinity binding of a phosphorylated C-terminal motif of receptor tyrosine kinase (RTK) (Stephens et al. 2005; Vadas et al. 2011). NORE1A’s activation displays features common to Raf (Liao et al. 2016). However, in this case, being a tumor suppressor in the Hippo pathway, it is the high affinity of the Sav-RASSF-Hippo (SARAH) heterodimer of NORE1A and mammalian sterile 20-like kinase 1/2 (MST1/2) that shifts the equilibrium toward an active NORE1A species. Ras binding releases NORE1A autoinhibition. Active NORE1A binding to MST1/2 increases the population of active MST kinase domain dimers.

Figuring out the autoinhibition and activation mechanisms of Ras targets at the membrane may not directly suggest Ras pharmacology; however, it can help clarify what may or may not work. Below, our review is within this light.

## Autoinhibition: variations on a theme

Autoinhibition and its release are common regulatory mechanisms, in solution, as for example in cyclic adenosine monophosphate (cAMP)- and cyclic guanosine monophosphate (c-GMP)-dependent protein kinases which are autoinhibited by interactions between their respective regulatory and catalytic domains (Francis et al. 2002), and in membrane-attached proteins, as in the neuronal membrane remodeling protein nervous wreck (NwK), which is autoinhibited by interactions between its membrane-binding Fes/Cip4 homology-Bin/Amphiphysin/Rvs167 (F-BAR) domain and its C-terminal Src homology 3 (SH3) domain (Stanishneva-Konovalova et al. 2016).

**Fig. 2:**
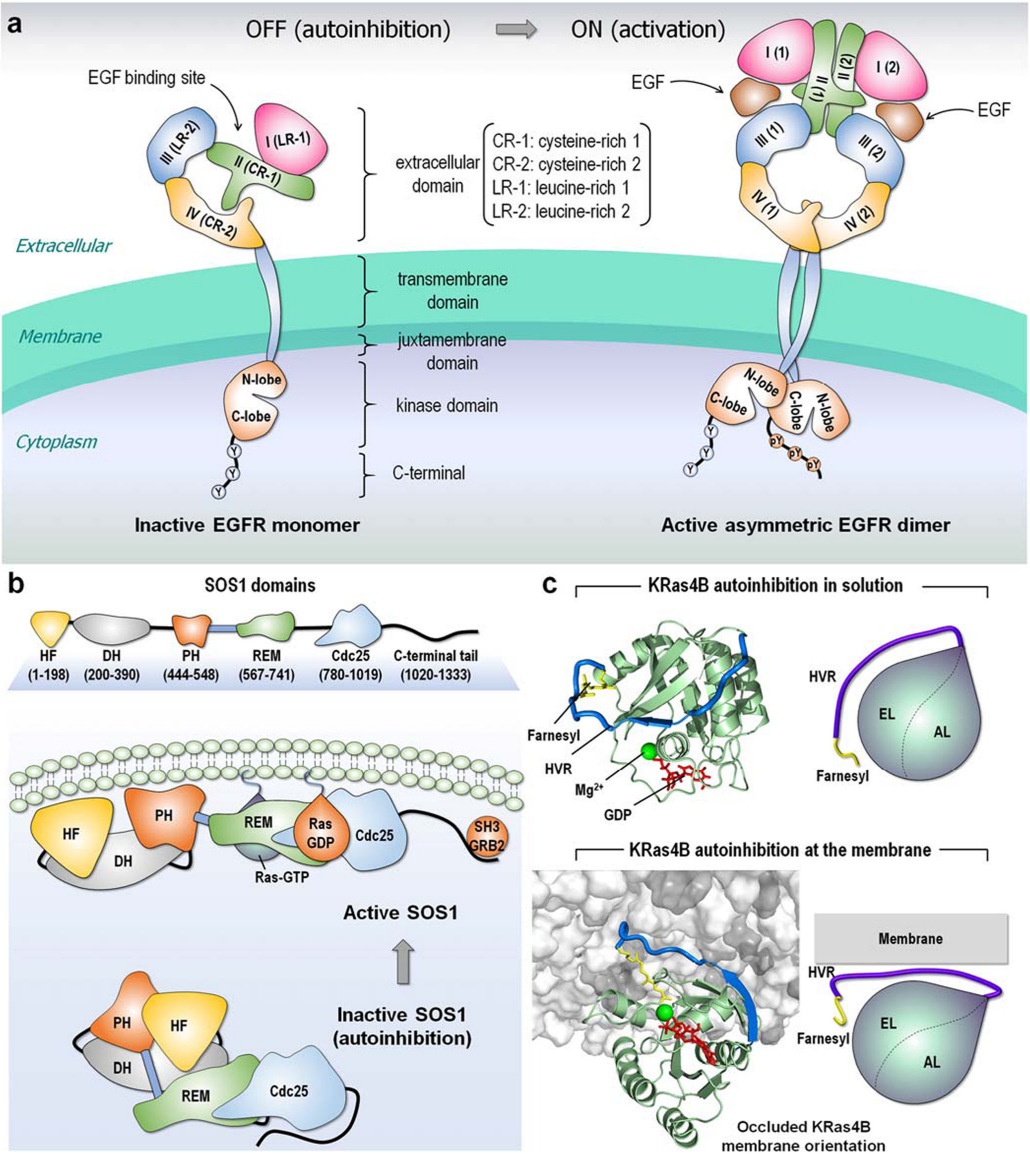
Examples of autoinhibition. **a** Collapsed conformation of the extracellular domain retains EGFR in an inactive, autoinhibition state. The inactive EGFR monomer can form a symmetric dimer but remains in the inactive state. The autoinhibition is released when the EGF ligand binds to the extracellular domain, causing an extended conformation of the extracellular domain and resulting in an asymmetric assembly of the kinase domains. This shifts the population to the active EGFR dimer. **b** SOS1, a GEF for Ras, contains histone-like fold (HF), Dbl-homology (DH), and pleckstrin-homology (PH) domains at the N-terminal region, and REM, Cdc25, and C-terminal SH3 binding motif at the C-terminal catalytic region. Inactive SOS1 is autoinhibited by the DH and PH domains that hamper the REM allosteric site for Ras binding. The C-terminal tail also blocks the Cdc25 catalytic site for Ras binding. The SH3 domain of growth factor receptor bound protein-2 (GRB2) binds the proline-rich SOS1 C-terminal tail and recruits SOS1 to the cell membrane. Membrane interactions of the DH and PH domains release autoinhibition and Ras association. **c** Autoinhibitions of KRas4B-GDP in solution and at the membrane interaction. In the inactive autoinhibition state, the HVR covers the effector binding site, resulting in occlusion of the catalytic domain membrane orientation. The KRas4B structures were adopted from previous work (Jang et al. 2016)

Autoinhibition is similarly common upstream and downstream Ras signaling cascades. Like all kinases, unbound epidermal growth factor receptors (EGFRs) are typically in the autoinhibited state with only 2 to 10% in the extended, active conformation (Schlessinger 2003). The EGFR tyrosine kinase domain is autoinhibited by intramolecular interactions between a short α-helix in its activation loop and the αC helix, which is shifted in active EGFRs, like Src family and cyclin-dependent kinases (CDKs) (Artim et al. 2012; Ferguson et al. 2003). In the extracellular domain of EGFR, the dimerization arms of subdomains II (cysteine-rich 1, CR-1) and IV (CR-2) interact, constraining subdomains I (leucine-rich 1, LR-1) and III (LR-2), preventing ligand binding (Schlessinger 2003). The high-affinity ligand binds to extended active state conformations, subdomain II dimerization arm is liberated, the equilibrium is shifted, relieving the autoinhibition and driving dimerization (Fig. 2a). Autoinhibition also regulates membrane-attached upstream Ras superfamily regulators (Cherfils and Zeghouf 2013). In guanine nucleotide exchange factors (GEFs), an active GTPase binds the allosteric site and mediates activation of an inactive molecule in the catalytic site, with a feed-forward loop flow. Son of Sevenless 1 (SOS1), which activates Ras, is one example (Fig. 2b) (Chardin et al. 1993; Cherfils and Chardin 1999; Gureasko et al. 2010; Lepri et al. 2011; Margarit et al. 2003; Rojas et al. 2011; Tartaglia et al. 2007; Vo et al. 2016). High affinity Ras-GTP binding at the allosteric site shifts the ensemble toward the active state, with large conformational changes (Boriack-Sjodin et al. 1998; Freedman et al. 2006; Pierre et al. 2011) that enable GDP exit and subsequent GTP binding (Liao et al. 2018). High affinity Ras-GTP binding is also the trigger for the population shift in KRas4B from an autoinhibited (GDP-bound) state, where the hypervariable region (HVR) loosely covers the active site (Fig. 2c), toward the active, open state (Chavan et al. 2015; Jang et al. 2015; Jang et al. 2016).

Autoinhibition is similarly common among Ras superfamily effectors, such as p21-activated kinase 1 (PAK1). PAK1 autoinhibitory domain quenches the catalytic kinase domain. Cdc42 high-affinity interaction activates PAK1 by binding to the same region as the regulatory domain. Conformational rearrangements and autophosphorylation release the autoinhibition (Deacon et al. 2008; Tu and Wigler 1999).

The advantages of autoinhibition are clear: it guards against spurious activation, setting a concentration threshold. The low affinity of the regulatory part permits signaling sensitivity. However, this challenges structural determination of the autoinhibited state, as in the cases of Raf, PI3Kα by both N- and C-terminal SH2 domains (nSH2 and cSH2), RASSF5 (NORE1A), and Ras. But relatively, weak interactions enable higher affinity activators, if present at sufficiently high effective local concentrations, to shift the equilibrium in favor of an “open” active state, executing the cell’s adaptation to the environment.

## Autoinhibition of Ras effectors

### Raf kinase

#### The autoinhibited state

**Fig. 3:**
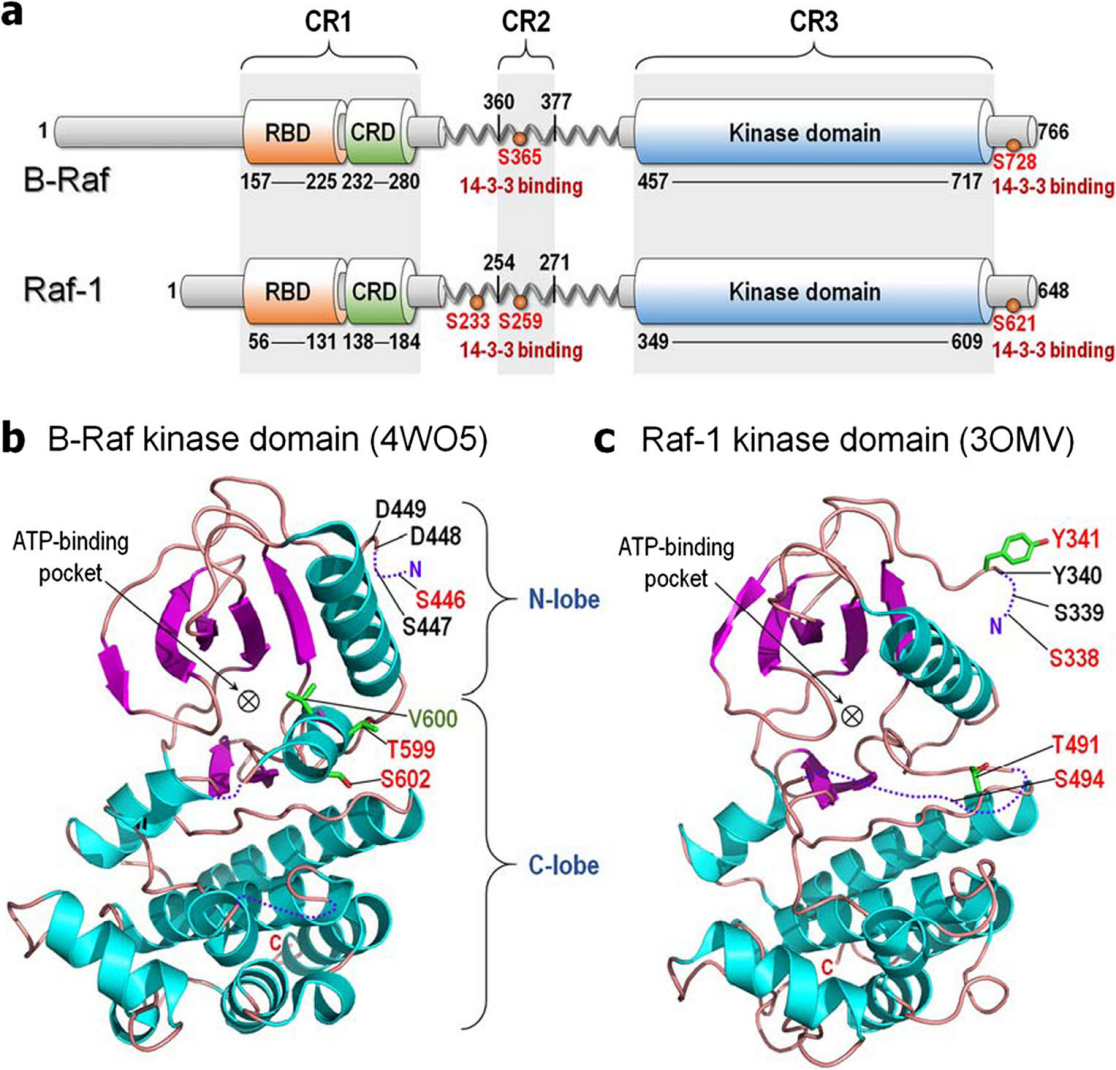
Structures of Raf kinase. **a** Domain structures of B-Raf and Raf-1. All Raf kinases share three conserved regions; CR1 involves the RBD and CRD, CR2 contains the Ser/Thr-rich region at the flexible linker, and CR3 is the kinase domain. For B-Raf, the 14-3-3 protein binds phosphorylated serine residues at Ser365 and Ser728 in CR2 and C-Terminal tail, respectively, and at Ser233, Ser259, and Ser621 for Raf-1. **b** Crystal structure of the kinase domain of B-Raf (PBD code: 4WO5). Phosphorylation at Ser446 weakens the autoinhibition, while S446A increases it. Acidic substitution and phosphorylation at Thr599 and Ser602 weaken the autoinhibition as well as the oncogenic mutation of V600E. **c** Crystal structure of the kinase domain of Raf-1 (PBD code: 3OMV). Phosphorylation at Ser338 and Tyr341, and acidic substitution and phosphorylation at Thr491 and Ser494 weaken the autoinhibition. Phosphorylated and mutated residues are marked red and green, respectively

The absence of a crystal (or NMR) structure of Raf’s autoinhibited conformation suggests an ensemble of “closed” monomeric states with the N-terminal tail, Ras binding domain (RBD), cysteine-rich domain (CRD), and linker, with its Ser/Thr-rich segment, hindering kinase domain dimerization. (The RBD-CRD region is denoted conserved region 1 (CR1) and the Ser/Thr-rich region as CR2 (Fig. 3a) (Lavoie and Therrien 2015; Terrell and Morrison 2018)). Several observations support such autoinhibiting organization:

(i). Removal of the N-terminal region dysregulates Raf’s kinase activity (Bruder et al. 1992; Fukui et al. 1987; Heidecker et al. 1990; Ishikawa et al. 1988; Ishikawa et al. 1986; Molders et al. 1985; Schultz et al. 1988; Stanton and Cooper 1987; Stanton et al. 1989);
(ii). Overexpression of CR1 abolishes Raf’s catalytic activity (Chong and Guan 2003; Cutler et al. 1998; Tran and Frost 2003; Tran et al. 2005);
(iii). B-Raf binding to active HRas abolishes the interaction of the N-terminal region with the kinase domain;
(iv). Ser446 phosphorylation of B-Raf weakens the autoinhibition but S446A substitution increases it, suggesting that alanine strengthens the interaction;
(v). Acidic substitutions at phosphorylation sites in the activation loop, Thr599 and Ser602 (Tran et al. 2005; Zhang and Guan 2000; Zhang and Guan 2001), and the oncogenic V600E mutation of B-Raf weaken the autoinhibition (Wan et al. 2004). These B-Raf activation loop residues in the kinase domain (Fig. 3b) (following the DFG motif (Kohler et al. 2016)) are analogous to Thr491 and Ser494 of Raf-1 (Fig. 3c) (Chong et al. 2001).
(vi). Phosphorylations of Ser338 and Tyr341 (in Raf-1; aspartic acids in B-Raf) weaken the autoinhibition (Chong and Guan 2003; Tran and Frost 2003);
(vii). B-Raf Ser446 (Ser338 in Raf-1) is constitutively phosphorylated in immortalized COS and PC12 cells (Mason et al. 1999; Tran et al. 2005). Further,
(viii). Phosphorylation of Ser259 (Raf-1) or Ser365 (B-Raf) in the CR2 by protein kinase A (PKA) (Cook and McCormick 1993; Dhillon et al. 2002b; Lavoie and Therrien 2015; Wu et al. 1993) and Akt (Rommel et al. 1999; Zimmermann and Moelling 1999) (or large tumor suppressor 1 (LATS1) (Romano et al. 2014)) affects autoinhibition (Lavoie and Therrien 2015), and S259A of Raf-1 promotes activation (Dhillon et al. 2002b; Morrison et al. 1993).

Finally, the importance of the region in autoinhibition is also implied by other nearby substitutions that enhance Ras–Raf binding (Dhillon et al. 2002a; Light et al. 2002). In line with this, phosphorylated Ser259 of Raf-1 is recognized by 14–3-3 proteins (Michaud et al. 1995; Muslin et al. 1996; Rommel et al. 1996; Tzivion et al. 1998), which also bind phosphorylated Ser621 (Fig. 3a) (Michaud et al. 1995; Muslin et al. 1996; Rommel et al. 1996). Concomitant 14-3-3 binding at both sites may stabilize the interaction between the N-terminal segment and the kinase domain (Matallanas et al. 2011; Tzivion et al. 1998). Simultaneous binding at phosphorylated Ser233 of Raf-1 was also observed (Dumaz and Marais 2003; Molzan and Ottmann 2012).

#### The active state

**Fig. 4:**
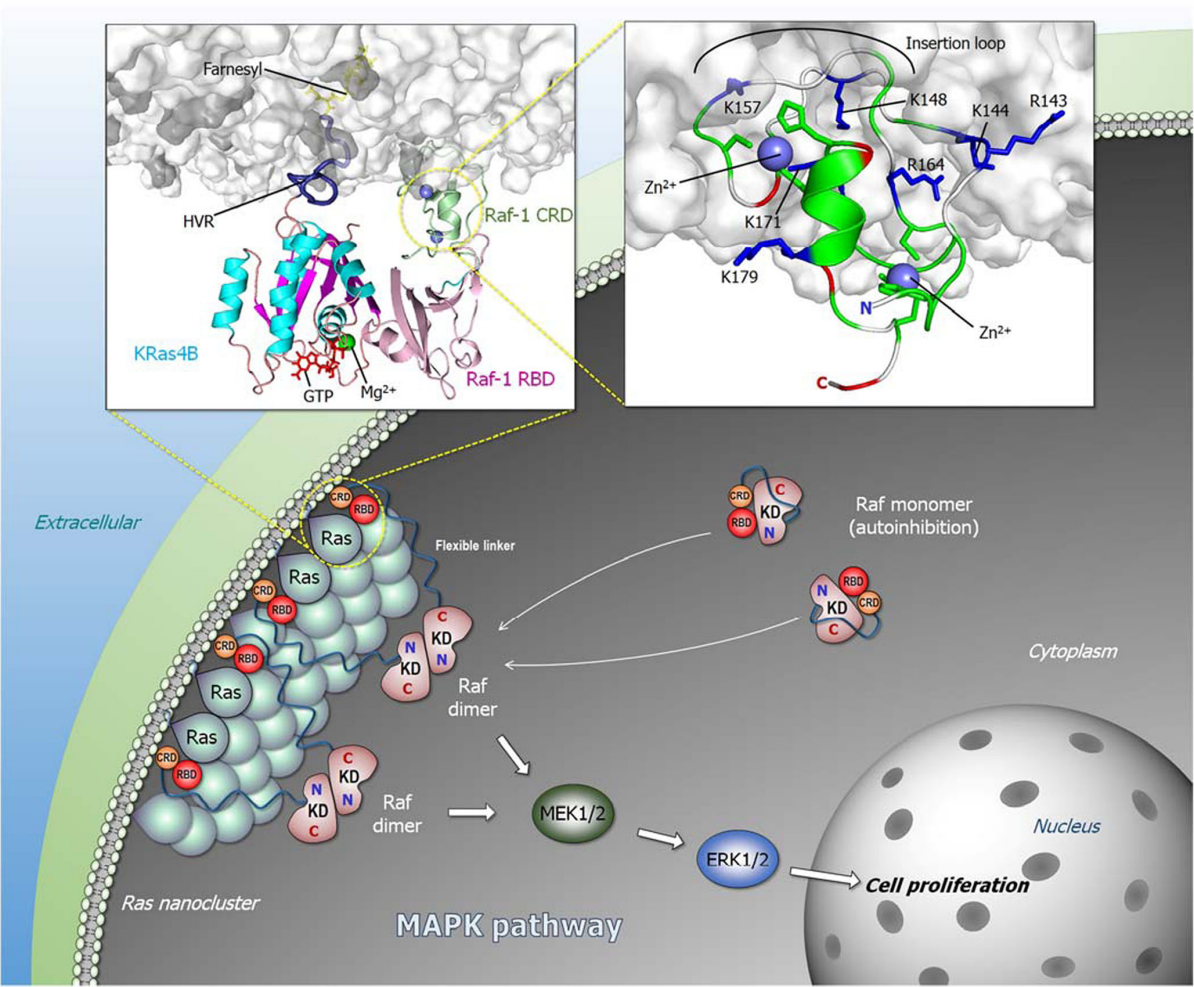
Ras/Raf signaling pathway. Ras forms nanoclusters and promotes Raf dimerization in the Raf/MEK/ERK (MAPK) pathway. Active Raf dimer tigers cascade phosphorylation signals through MEK1/2 to ERK1/2, leading to cell proliferation. The left inset cartoon represents the organization of KRas4B–RBD-CRD of Raf-1 complex at the membrane. CRD reduces Ras–RBD fluctuations at the membrane, increasing Ras–RBD affinity. The right inset cartoon shows the membrane interaction of Raf-1 CRD with the highlighted key basic residues (blue sticks) and the insertion loop containing hydrophobic residues (white cartoon tube). Zn^2+^ coordination sites are highlighted by green sticks. The cartoons were depicted from previous simulations (Li et al. 2018a)

Whereas structural data are unavailable for Raf’s autoinhibited state, some NMR and crystal structure data, coupled with modeling are available for the Ras-bound state (Fig. 1a). The high affinity (nanomolar range) binding of Raf’s RBD to active Ras recruits Raf to the plasma membrane (Chuang et al. 1994; Herrmann et al. 1995). The crystal structure of Ras with RBD is available (Fetics et al. 2015), as well as NMR structural data of CRD in solution (Mott et al. 1996). Abundant data point to Ras proteins anchorage via their prenylated HVR, and their spatial proximity—nanoclusters or dimers/oligomers—increases Raf’s effective local concentration, promoting the dimerization of Raf’s kinase domains and activation (Fig. 4). However, even though already early on CRD was established to interact with the membrane and stabilize the Ras–Raf interaction (Chuang et al. 1994), how it fits into Raf activation scenario and exactly what is its function have been unclear and challenging to address experimentally (Li et al. 2018a). Three recent modeling publications (Li et al. 2018a; Li et al. 2018b; Travers et al. 2018) aimed to elucidate its role. Isolated Raf-1 CRD can stably anchor to the membrane. This occurs via basic residues interjecting into the amphipathic membrane surface (right inset of Fig. 4). Explicit solvent molecular dynamics simulations suggest that in Raf-1 this “membrane insertion” loop region residues Lys144, Lys148, and Lys157 play a key role in the membrane attachment (Li et al. 2018a), which is supported by experimental mutational data of Arg143, Lys144, and Ly148 (Improta-Brears et al. 1999). Hydrophobic residues in the loop further enhance the membrane attachment. Modeling points to a high probability of the loop interacting with the membrane, particularly phosphatidylserine (Li et al. 2018a), consistent with experimental observations (Improta-Brears et al. 1999). Significantly, simulations of the KRas4B–Raf-1 RBD-CRD (left inset of Fig. 4) also suggest that the preferred conformation and orientation of the CRD resembles that favored by the CRD when modeled only with the membrane (Li et al. 2018a). In Raf-1, the linker between Raf RBD and CRD is short; only 6 residues. The short linker connecting CRD to the RBD confines its spatial location in the complex between the RBD and the membrane, with no detected interactions of CRD with the KRas4B catalytic domain. Whereas the HRas farnesyl group can interact with the Raf-1 CRD (Thapar et al. 2004), while the two palmitoyls retain the Ras anchorage in the membrane, this is not the case in KRas4B where the farnesyl remains anchored. Jointly, the membrane-anchored HVR and the CRD can diminish the fluctuations of the Ras–RBD complex at the membrane, which increases its affinity. The reduced fluctuations stabilize the Ras–Raf RBD interaction, which promotes Raf activation. The details of exactly how Ras orients at the membrane, e.g., α3/α4 or α4/α5, the angle with respect to the membrane normal, and whether these reflect Ras isoform-specific preferences, which is what we believe, are still controversial (Abankwa et al. 2010; Gorfe et al. 2007; Jang et al. 2016; Li and Buck 2017; Mazhab-Jafari et al. 2015); reviewed in the literature (Li et al. 2018a). However, key considerations are the populations, or residence times of the different organizations and the availability of Ras effector binding sites for the RBD interaction.

Considering the high flexibility of the system, multiple organizations of Ras–RBD-CRD at the membrane are possible. The cartoon (left inset of Fig. 4) depicts those of KRas4B–Raf-1 which we observed to be among the most favored (Li et al. 2018a). In these orientations, the effector binding site of Ras faces away from the membrane surface allowing the RBD to interact with the Ras catalytic domain and the CRD to interact with the membrane. Ras is anchored in the membrane through its prenylated HVR. Even though the Ras–RBD interaction is of high affinity in solution, simulations indicate that at the membrane the fluctuations are significant. The constrained positioning of the CRD, contacting the membrane through the basic “insertion loop” and further supported by hydrophobic residues, and restricted by the short linker, restrains the fluctuations and stabilizes the Ras–RBD interaction. The observation that this orientation resembles that populated by CRD in the absence of the Ras–RBD lends support to this organization. Notably, CRD can recruit Raf-1 to the membrane even in the absence of Ras; however, by quenching the KRas4B–Raf-1 RBD fluctuations, CRD stabilizes the interaction, which increases the population of the active Raf species and thus signaling.

#### The activation mechanism

Even though the Ras–Raf link in mitogen-activated protein kinase (MAPK) has been known for decades, exactly how Ras activates Raf has been puzzling. Over the last few years, the community focused on the kinase domain dimer and its structure with the aim of unraveling the mechanism of the autophosphorylation, why and how resistance can defeat Raf inhibitors interacting with the kinase domains and overcoming the dimerization requirement, and broadly drug discovery (Freeman et al. 2013; Hatzivassiliou et al. 2010; Hu et al. 2013; Jambrina et al. 2014; Jambrina et al. 2016; Lavoie and Therrien 2015; Lavoie et al. 2013; Poulikakos et al. 2010; Rajakulendran et al. 2009; Thevakumaran et al. 2015; Zhang and Guan 2000; Zhang et al. 2015). However, much less was revealed about activation scenarios at the membrane. Possible reasons for this include the challenging presence of the long linker (~ 180 residues in B-Raf; ~ 170 residues in Raf-1) between CRD and the kinase domain (Fig. 3a), and the attachment to the membrane. In addition, the likely relatively weak interaction between the regulatory N-terminal region and the kinase domain may also be unyielding to experimental structural determination. Nonetheless, data have accumulated providing some insight into the inactive autoinhibited state, and thus the activation mechanism (Fig. 4).

In drawing a possible scheme (that awaits testing), we consider the high affinity of the Ras–RBD interaction: the residues whose phosphorylation abolishes the autoinhibition, data related to the consequences of alanine substitution, the suggested binding of the 14-3-3 protein to two phosphorylated sites as described above (Dumaz and Marais 2003; Lavoie and Therrien 2015; Molzan and Ottmann 2012; Tran et al. 2005), and our expectation that the inhibitory N-terminal is not tightly bound to the kinase domain. Rather than a hierarchical step-by-step activation process, we favor an ensemble view of the mechanism. The fundamentals of this concept, as well as its utilization to explain a broad range of observations have already been discussed (Boehr et al. 2009; del Sol et al. 2009; Kumar et al. 2000; Liu and Nussinov 2016; Liu and Nussinov 2017; Ma et al. 1999; Nussinov 2016; Nussinov et al. 2011; Nussinov et al. 2013c; Nussinov and Wolynes 2014; Tsai et al. 2009; Tsai et al. 1999a; Tsai et al. 1999b; Tsai and Nussinov 2014b; Wei et al. 2016). Raf molecules exist in an ensemble of conformations which includes all autoinhibited (inactive) species and the active state; however, the distribution of the ensemble depends on the conditions; that is, whether the cell is in a resting state, or stimulated/oncogenic. In the absence of an active, GTP-bound membrane-anchored Ras molecule, wild type Raf largely populates an autoinhibited state (Fig. 4), with a minor component of the active, “open” state. In the autoinhibited state, those segments pointed out by mutational and phosphorylation data may interact with the kinase domain. That this interaction may not be sufficiently stable is also suggested by the dual 14-3-3 interaction. In the presence of active Ras (and other cell factors possibly interacting with 14-3-3, as well as dephosphorylation of these sites, e.g., by protein phosphatase 2A (PP2A) and protein phosphatase 1 (PP1), (Abraham et al. 2000; Dhillon et al. 2002a; Jaumot and Hancock 2001; Lavoie and Therrien 2015; Ory et al. 2003)), the equilibrium shifts toward the uninhibited “open” state. The tight interaction with Ras is expected to alter the distribution of the ensemble, favoring the released intramolecular interactions with the kinase domain. This would explain *why* Raf evolved this high-affinity interaction. Phosphorylation events further contribute to the shift of the ensemble toward the active state (Nussinov et al. 2012). We note that such an ensemble view does not favor a description where an interaction actively “opens” Raf; instead, it advocates preexistence of states whose relative populations change with cell events, such as phosphorylation (or dephosphosphorylation), proximity of active Ras molecules, etc. This view is based on fundamental physicochemical principles. Macromolecules are not solid rocks; they are flexible, visiting multiple possible conformations (Nussinov and Wolynes 2014). Exactly which N-terminal and kinase domain regions interact, and whether these overlap the interaction with Ras, awaits modeling and data, such as NMR-detected residue-specific chemical shift perturbations, and high-resolution cryo-EM electron density maps. We believe that such data will point to the presence of several possible states.

#### Insight into drug discovery

Is blocking the release of the autoinhibition a productive strategy in drug discovery? Despite the absence of direct structural data of the autoinhibited state, the flexibility and the related relatively weak affinity of the intramolecular N-terminal region-kinase domain interaction underscore the challenge in targeting it. In agreement with these considerations, recently, the Therrien team successfully obtained Raf inhibitors that shift the equilibrium toward a *disrupted* autoinhibited state (Jin et al. 2017). Introduction of the inhibitor resulted in interruption of the presumably unstable intramolecular interactions between the N-terminal regulatory region and the kinase domain, independently of Ras state. Thus, to effectively disrupt the autoinhibitory interactions, high-binding affinity would be required, resembling Ras’ mode of action. A compound that would stabilize the autoinhibited state would be very difficult to attain.

### PI3Kα lipid kinase

#### There are two independent components in PI3Kα activation

**Fig. 5:**
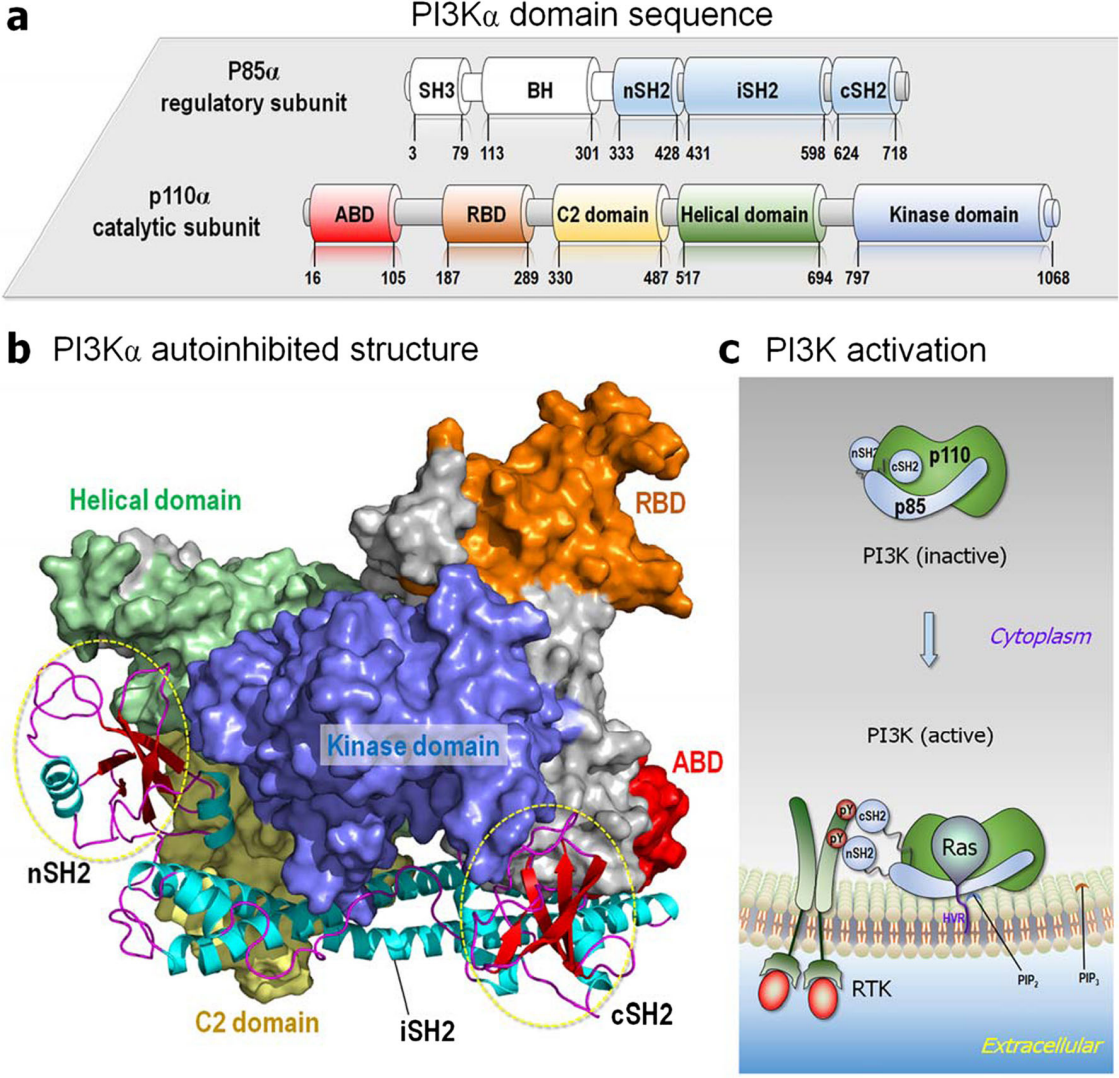
Domain structure of PI3Kα. **a** PI3Kα is composed of p110α catalytic and p85α regulatory subunits. While the structure of the p110α subunit has been determined, no structure is currently available for the N-terminal region of the regulatory subunit (SH3 and BH domains, denoted as white cylinder). **b** Modeled structure of PI3Kα, which is constructed from the crystal structure (PDB code: 4OVV), represents an autoinhibition structure. The interactions of the nSH2 and cSH2 domains of p85α with the catalytic p110α subunit impede PI3Kα activation. **c** Phosphorylated pYXXM motif (denoted as pY) of RTK liberates the nSH2 and cSH2 domains from the catalytic subunit, removing autoinhibition in the catalytic subunit and thus exposing the active site

The regulation of PI3Kα also involves autoinhibition; however, the scenario at the membrane differs (Nakhaeizadeh et al. 2016). PI3Kα produces phosphatidylinositol (3,4,5)-trisphosphate (PIP_3_) from phosphatidylinositol 4,5-bisphosphate (PIP_2_), a critical signaling molecule that stimulates the PI3K/Akt/mTOR (mammalian target of rapamycin) pathway, which regulates cell growth and survival, cytoskeleton reorganization, and metabolism (Castellano and Downward 2011). PI3Kα consists of regulatory (p85α) and catalytic (p110α) subunits (Fig. 5a). The catalytic subunit contains five domains: the adaptor binding domain (ABD) that binds p85, the RBD, the C2 membrane-binding domain, the helical domain, and the catalytic kinase domain. The regulatory subunit consists of two SH2 domains (nSH2 and cSH2) separated by an intervening coiled-coiled domain (iSH2) (Nussinov et al. 2017b; Nussinov et al. 2018d; Zhang et al. 2017). The interactions of these SH2 domains with p110 result in covering PI3Kα active site (Fig. 5b), hindering the binding of the PIP_2_ substrate. PI3Kα becomes fully activated by binding to an active, GTP-bound Ras, and to phosphorylated C-terminal motifs (pYXXM) of RTKs, such as EGFR (or an associated protein, e.g., insulin receptor substrate 1 (IRS-1) (Backer et al. 1992)). The motifs of the RTK molecules interact with the nSH2 and cSH2 domains (Fig. 5c). The high-affinity binding of the phosphorylated C-terminal RTK motifs releases the autoinhibitory interactions primarily of nSH2, but also of cSH2 with the catalytic subunit, resulting in exposed active site (Nussinov et al. 2015a; Nussinov et al. 2016a; Nussinov et al. 2017b). The release of the autoinhibitory nSH2 domain allosterically helps PI3Kα activation. RBD’s binding to active Ras contributes by recruiting PI3Kα to the membrane and to allosteric activation of the kinase domain. Both events, binding the pYXXM motifs and Ras, increase the accessibility of PI3Kα to the PIP_2_ substrate at the membrane (Nussinov et al. 2016a).

The mechanisms of oncogenic mutations validate the two independent components in PI3Kα activation mechanism. Autoinhibition can be relieved by the E542K and E453K mutations in the helical and C2 domains, respectively. These mutations replace the stabilizing salt-bridge interactions of the nSH2 domain with the catalytic subunit by charge repulsion (Burke et al. 2012; Gabelli et al. 2014; Mandelker et al. 2009). N345K is another mutation that disrupts favorable inhibitory wild-type interaction. This mutation is at the iSH2-C2 interface in the C2 domain. The weakened interaction leads to loss of structural coupling to the nSH2 domain, loosening its autoinhibition (Gabelli et al. 2014), and to more favorable membrane binding, as indicated by an increased hydrogen/deuterium exchange level (Burke et al. 2012). Membrane binding is allosterically coupled to the ABD-RBD linker far away (Ma et al. 2011). Further, the R88Q mutation in the ABD abolishes the hydrogen bonding of the arginine with the kinase domain (Zhao and Vogt 2008a). The resulting higher catalytic activity is consistent with another mutation involving a deletion of the ABD (Zhao and Vogt 2010), pointing to the ABD as part of the nSH2 autoinhibition via association with the catalytic subunit. On the other hand, the allosteric stimulation by active Ras binding to p110 RBD (Gupta et al. 2007; Kodaki et al. 1994) resembles conformational changes in the p110 C-lobe at the membrane interface induced by PI3Kα “hot spot” (H1047R) mutation in the kinase domain of p110 (Mandelker et al. 2009; Zhao and Vogt 2008b). The resulting increased membrane binding and accessibility to PIP_2_ makes the H1047R mutational variant independent of Ras-GTP (Zhao and Vogt 2008b). Thus, whether in Raf or in PI3Kα, oncogenic mutations can inform about activation mechanisms.

#### PI3Kα activation scenario at the membrane

Thus, unlike Raf whose activation at the membrane involves a shift of the ensemble toward the more stable Ras–Raf interaction (Fig. 4), PI3Kα activation at the membrane involves two independent events: release of the autoinhibition by RTK’s phosphorylated C-terminal motif and Ras allosteric activation (Fig. 5c). Both are required for full activation. Ras isoforms do not display high affinity to PI3Kγ, which is much lower than PI3Kα, but is sufficient for interactions of two proteins (Pacold et al. 2000; Rodriguez-Viciana et al. 1996). This micromolar affinity interaction with Ras already indicates that its role is unlikely to be mediated via a population shift of the autoinhibited state. At the same time, the PI3Kα substrate is anchored at the membrane, indicating that the PI3Kα active site must be at the membrane and oriented for PIP_2_ to dock into it. Like Raf, where the autoinhibitory interactions are relatively weak, in PI3Kα, the interactions of the SH2 domains, especially cSH2, with the kinase domain are also unstable; however, these are released via the involvement of the RTK motif. Further in contrast to Raf, even though PI3K is a dimer, it essentially acts as a monomeric unit. As such it does not require Ras nanoclustering (or dimerization). Finally, the two-component activation explains why even without Ras, PI3Kα can be activated—albeit not fully.

The dissociation constant, *K*_D_, for the Ras–PI3K complex is higher than that for the Ras–Raf RBD (Herrmann et al. 1995; Sydor et al. 1998) and the Ras–RBD of Ras-Ral guanine nucleotide dissociation stimulator (RalGDS) complexes (Herrmann et al. 1996), underscoring the role of calmodulin (CaM) in PI3K activation, and the different mechanisms of activation at the membrane. Thus, as we discuss below, even though both effectors deploy autoinhibition as a key component in their regulation, their detailed mechanisms differ, as do their chemical reactions. PI3Kα lipid kinase catalytic activation is at the membrane, which is not the case for Raf’s protein kinase domains. The exposed active site of the lipid kinase domain needs to be productively oriented toward the membrane. The PIP_2_ substrate is membrane-anchored and phosphorylated at the active site. The solvent-miscible ATP is far away.

#### PI3Kα catalysis

Raf is a protein kinase. Its substrates are proteins. By contrast, PI3Kα phosphorylates lipids. Even though there is some similarity in their active sites—both bind ATP—the different types of substrates and environments evolved distinctiveness. PI3Kα catalysis is regulated by membrane binding and the population of effective phosphate transfer transition complexes (Burke et al. 2012; Gabelli et al. 2014; Nussinov et al. 2017b; Zhao and Vogt 2008a). Experimental data indicate that both are required for full activity, and that they are structurally coupled. This can be seen by the *K*_m_, the PIP_2_ concentration at which PI3Kα is at half of its maximal catalytic rate (*k*_cat_). The Michaelis-Menten kinetic efficiency (*k*_cat_/*K*_m_) directly measures the enzyme activity. Here, the dissociation of nSH2 from p110α enables the effective formation of phosphate transfer transition complex, analogous to an increase of *k*_cat_. Experimental data indicate that both membrane binding capability and effective formation of the phosphate transfer transition complex are required for a fully active PI3Kα (Nussinov et al. 2015a). PI3Kα activity reflects the efficiency of the individual steps, ATP and PIP_2_ (cofactor and substrate, respectively) binding, phosphoryl transfer, and dissociation of the products. If we do not consider ATP binding and ADP and PIP_3_ dissociation steps, activation can be evaluated by *k*_cat_/*K*_m_ based on a two-step reaction (Miled et al. 2007). PIP_2_ accessibility to the PI3Kα active site is connected to its ability to bind the membrane, as indicated in the *K*_m_. By contrast, the release of the autoinhibition by the dissociation of the nSH2 from the catalytic subunit effectively permits the formation of phosphate transfer transition complex, correlates with an increase of *k*_cat_.

#### Insight into drug discovery

To date, there are no PI3Kα drugs in the clinics. Exploiting the autoinhibited states may be challenging. However, currently, we are exploring strategies to obstruct the kinase reaching a catalytically-productive state involving the ATP and the lipid substrate at the membrane, a scenario differing from that of Raf.

### NORE1A tumor suppressor

#### NORE1A: autoinhibition and its release

**Fig. 6:**
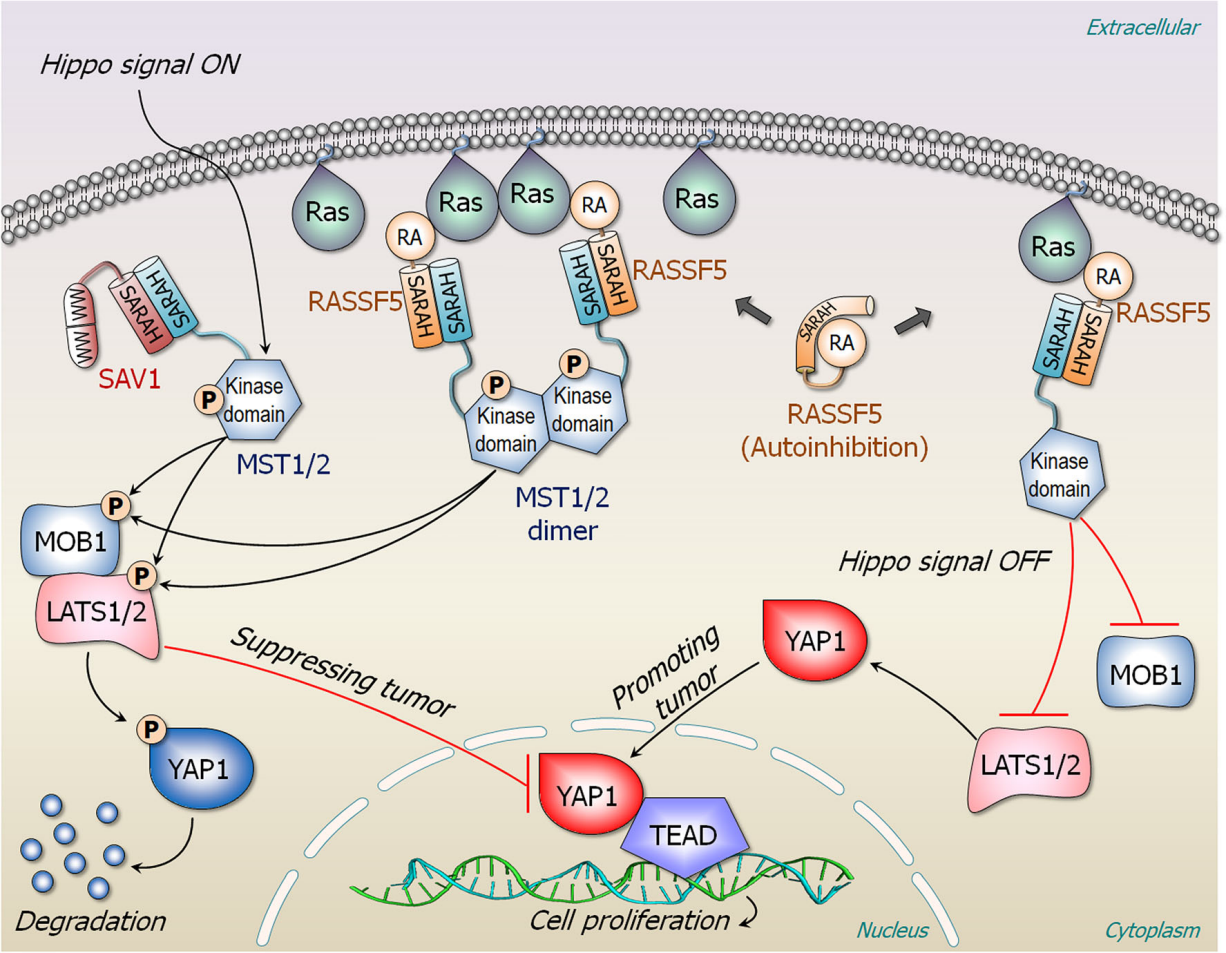
RASSF5 (NORE1A) and Hippo pathway. RASSF5 is an adaptor protein and composed of the cysteine-rich C1 (not shown), RA, and the SARAH domains. An isolated RASSF5 is autoinhibited by its RA domain interacting with its SARAH domain. In the presence of Hippo signal, MST1/2 binds to salvador homolog 1 (SAV1) scaffolding protein and is phosphorylated by upstream signal. SAV1 contains two consecutive Trp-rich WW domain and the SARAH domain. The interaction of SAV1–MST1/2 promotes phosphorylation of MOB kinase activator 1 (MOB1) and LATS1/2. A phosphorylation cascade to YAP1 causes degradation of p-YAP1 and thus suppressing tumor. In the presence of proximal Ras nanocluster, two RASSF5 proteins bind a Ras dimer, liberating their SARAHs to recruit MST1/2 SARAH, facilitating MST1/2 dimerization through their kinase domains. Cross phosphorylated MST1/2 by each kinase domain promotes phosphorylation of MOB1, LATS1/2, and YAP1, resulting in its degradation. In the absence of Hippo signal, the unphosphorylated YAP1 can be translocated into the nucleus and bind to the transcription factor TEAD. This leads to cell proliferation as the MAPK pathway. Without proximal Ras nanocluster, RASSF5 in complex with monomeric Ras does not promote MST1/2 dimerization and phosphorylation, increasing population of unphosphorylated YAP1

NORE1A (RASSF5) interacts with active Ras and activates the MST1/2 (Avruch et al. 2009; Avruch et al. 2012; Donninger et al. 2016; Liao et al. 2016; Nussinov et al. 2017a; Richter et al. 2009). The signal propagates through the Hippo-pathway phosphorylation cascade, leading to phosphorylation of Yes-associated protein 1 (YAP1), which targets it for degradation (Fig. 6). Overexpressed YAP1 promotes cell proliferation (Fallahi et al. 2016). NORE1A, Raf, and PI3K all share the same binding site on Ras. However, different from these other Ras effectors, NORE1A is not a kinase. In its inactive state, NORE1A is autoinhibited by its Ras association (RA) domain interacting with its SARAH domain. The two domains are connected by a flexible hinge. The interaction is weak, and fluctuating; thus, no experimental structure. When activated, the SARAH domain swings to dissociate from the RA domain and heterodimerize with the MST1/2 SARAH domain. In the MST inactive state, the kinase domain is autoinhibited by its SARAH domain; in the open state, the SARAH is dissociated from the kinase domain. The high-affinity SARAH heterodimer (of the NORE1A and the MST1/2) interaction shifts the equilibrium toward the active open MST1/2 state, enabling the homodimerization of the kinase domains, and the trans-autophosphorylation. Even though MST1/2 SARAH homodimerization can take place, its affinity is lower than that of the hetero-SARAH dimer (Hwang et al. 2014; Makbul et al. 2013). Thus, mechanistically, NORE1A plus MST1/2 resemble Raf. As in Raf, Ras dimer (or nanocluster) can associate with two NORE1A molecules, which heterodimerize with two MST’s SARAH domains (Fig. 6). However, the functional outcome differs: Raf promotes proliferation whereas NORE1A suppresses it. MST1/2 requires NORE1A’s help since it does not have a Ras-binding domain. NORE1A is essentially an adaptor bridging Ras and MST (Liao et al. 2016). The Ras interaction is essential, since it brings the two MST1/2 kinase domains into spatial proximity, promoting MST1/2 activation (Liao et al. 2017; Stieglitz et al. 2008). In its absence, the effective local concentration may be too low. NORE1A binds to Ras through its RA domain with a micromolar affinity. Thus, whereas the tight interaction between Raf’s RBD and Ras drives the equilibrium toward Raf’s open active state, for NORE1A, the high-affinity interaction driving the active MST1/2 kinase state is that between the two SARAH domains. Its C1 domain anchors into the membrane. Its exact role in the mechanism of NORE1A activation is still unclear, although it is possible that is resembles that of Raf’s CRD. Through the NORE1A–MST SARAH domain heterodimerization, the MST kinase domain links to active Ras, inducing the MST kinase domain dimerization and phosphorylation just like Raf’s kinase domain (Liao et al. 2016).

The micromolar range RA–Ras interaction is insufficient to drive the equilibrium in the way that the nanomolar Raf RBD–Ras does. However, the high affinity SARAH NORE1A–MST1/2 heterodomain interaction can, shifting the equilibrium toward MST1/2 kinase domain homodimerization and activation. Thus, even though there is a general mechanistic similarity between NORE1A + MST1/2 and Raf, and the release of the autoinhibition through a shift of the ensemble is caused by a high-affinity interaction, the details differ: whereas in Raf the interaction with Ras is the cause, in NORE1A the cause is the SARAH domain interaction with MST1/2. Either way, in both cases, the outcome is the dimerization and activation of the kinase domains.

#### Insight into drug discovery

In the case of RASSF5 tumor suppressor, pharmacological efforts should not be aimed at promoting the autoinhibition but abolishing it.

## Additional factors in activation at the membrane

### Overview

Mutations and additional cell-specific factors can also play a role in effector activation. These may relate to, e.g., expression, cell-to-cell variation in gene expression and the propagation of such variation (Martins et al. 2017), plasma membrane microdomain environment (Goldfinger and Michael 2017; Lommerse et al. 2005; Zhang et al. 2018a), membrane-associated scaffolding proteins (Michael et al. 2016), membrane delivery (Weise et al. 2011), and ubiquitination/deubiquitination (Simicek et al. 2013; Wan et al. 2017) which may affect HVR-membrane attachment, thus Raf versus PI3K signaling, as well as sumoylation (Choi et al. 2018). Mutations may also differentially affect Ras target activation (Hamad et al. 2002; Nussinov et al. 2015b; Rodriguez-Viciana et al. 2004; Rodriguez-Viciana et al. 1997; Vandal et al. 2014). KRas^T35S^ and KRas^D38E^ activate MAPK signaling, but do not bind RalGDS or PI3K; however, KRas^G12V/T35S^ or KRas^G12V/D38E^ mutants are associated with higher cancer occurrence than KRas^G12V^ and KRas^G12V/E37G^. KRas^G12V/E37G^ prefers RA domain-containing proteins such as NORE1A more than other mutants (Chang et al. 2014; Schubbert et al. 2007; Vandal et al. 2014). Further, KRas^G12V/Y40C^ coexpressed with B-Raf^V600E^, appears to be less oncogenic than with B-Raf^V600E^ expression alone. Expression of B-Raf^V600E^ and KRas^G12V/D38E^ resulted in smaller tumors than of only B-Raf^V600E^. KRas^T35S^, but not KRas^D38E^, interacts weakly with RalGDS and Ral GDP dissociation stimulator-like (RGL) (Rodriguez-Viciana et al. 2004; Rodriguez-Viciana et al. 1997). Preferred effector interaction may reflect shifts in the conformational ensemble by specific mutations, or combinations (Nussinov et al. 2013b; Nussinov and Tsai 2015; Tsai and Nussinov 2014a; Tsai and Nussinov 2014b). Below, we briefly focus on two factors, CaM and scaffolding proteins.

### Calmodulin

**Fig. 7:**
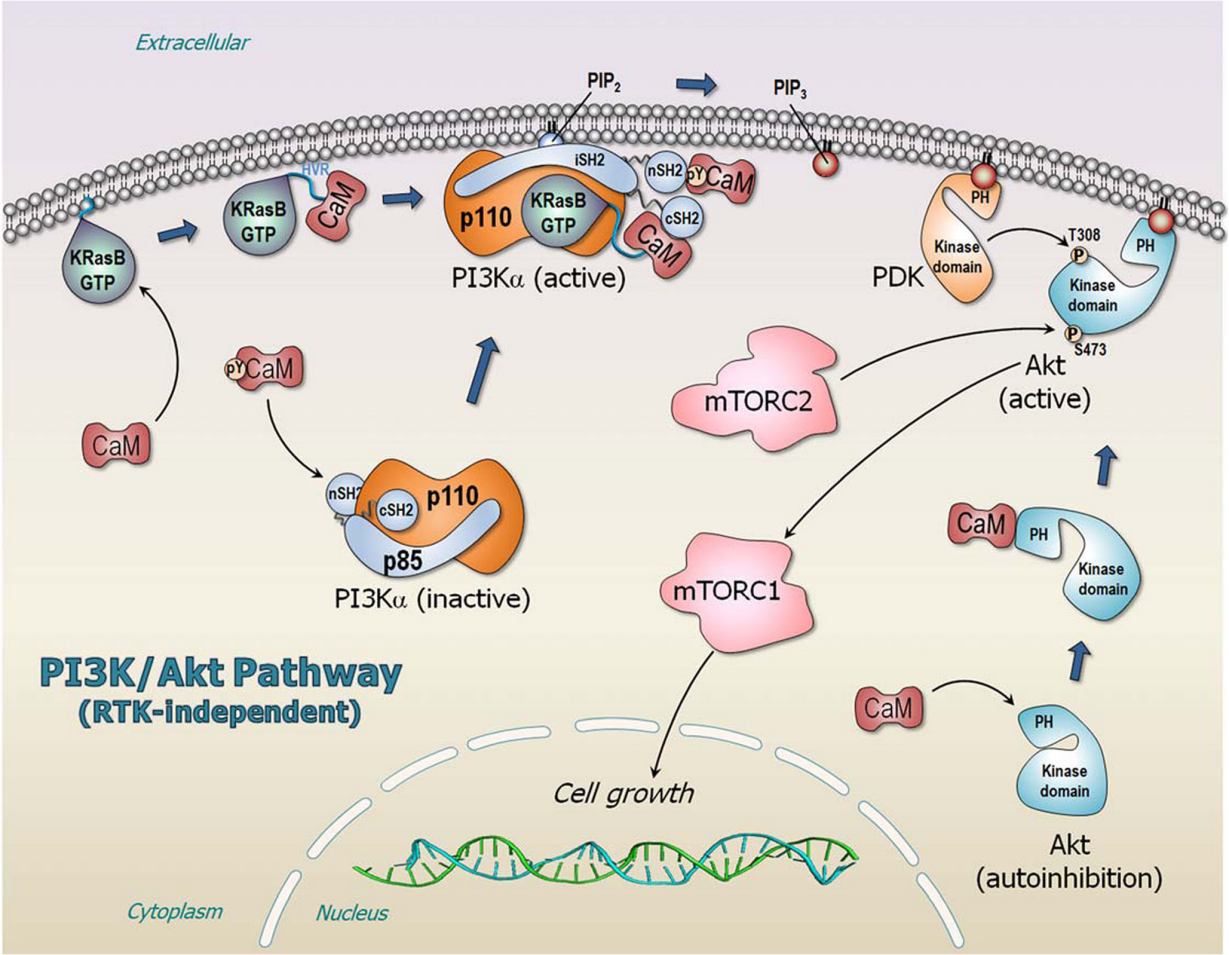
RTK-independent PI3Kα/Akt/mTOR pathway leading to cell growth. In adenocarcinoma of *KRAS*-driven cancer, CaM specifically recruits active KRas4B from the membrane to PI3Kα and activates it. The binding site of CaM with phosphorylation at Tyr99 (pY99-CaM) acts as the RTK’s pYXXM motif, substituting for the missing RTK signal. Interactions of CaM with n/cSH2 domains of p85α regulatory subunit remove the autoinhibition on the p110α catalytic subunit. CaM also removes the autoinhibition of Akt, releasing the PH domain from its kinase domain, and recruits Akt to the plasma membrane promoting the PH domain to bind PIP_3_. PI3Kα phosphorylates PIP_2_ to produce PIP_3_ that recruits both PDK and Akt to the plasma membrane

Oncogenic Ras can bind PI3Kα’s RBD and recruit the kinase to the membrane, but it is unable to fully activate it. In the absence of the signal-activated phosphorylated RTK’s C-terminal motif, the autoinhibition is still in place. Based on experimental data (Chaudhuri et al. 2016; Joyal et al. 1997; Joyal et al. 1996; Liao et al. 2006), we suggested that Ca^2+^-CaM, especially when phosphorylated at Tyr99, can complement KRas (KRas4A and KRas4B) by binding to the n/cSH2 domains, replacing the missing RTK signal (Fig. 7) (Nussinov et al. 2015a; Nussinov et al. 2017b; Wang et al. 2018; Zhang et al. 2017; Zhang et al. 2018b). CaM’s negatively-charged linker binds tightly to KRas’ highly positively charged HVR (Abraham et al. 2009; Jang et al. 2017), and its hydrophobic N-lobe pocket offers a docking site to the KRas farnesyl group (Banerjee et al. 2016; Jang et al. 2017); but is unable to stably bind the neutral HVRs of HRas and NRas (Alvarez-Moya et al. 2011; Villalonga et al. 2001). This can explain CaM’s role in *KRAS*-driven cancers (Nussinov et al. 2017b), in line with genetically-engineered mouse models showing that oncogenic KRas can induce senescence or proliferation and differentiation (Xu et al. 2014), but is unable to induce full PI3Kα activation. Our structural model of the PI3Kα heterodimer (Vadas et al. 2011; Vanhaesebroeck et al. 2001) includes the p110α catalytic subunit and the three p85α n/i/cSH2 domains (Fig. 5b), ATP in the p110 cleft between the N- and C-lobes, the two phosphorylated RTK peptides binding the n/cSH2 (Nussinov et al. 2015a), and PIP_2_ at the p110 active site. Different proteins can fulfill CaM’s role in oncogenic HRas and NRas in full activation of PI3Kα (Nussinov et al. 2018d), with scaffolding protein, IQ motif containing GTPase activating protein 1 (IQGAP1) being one possibility (Hedman et al. 2015; Ren et al. 2008). Direct physical interaction between Raf kinase and oncogenic Ras promotes Raf side-to-side dimerization (Fig. 4) (Rajakulendran et al. 2009) and MAPK signaling (Crews and Erikson 1993); however, both oncogenic KRas4B and CaM are involved in PI3Kα and Akt activation (Fig. 7), with CaM directly activating PI3Kα (Joyal et al. 1997; Liao et al. 2006), as well as Akt (Agamasu et al. 2015; Agamasu et al. 2017).

### Scaffolding

**Fig. 8:**
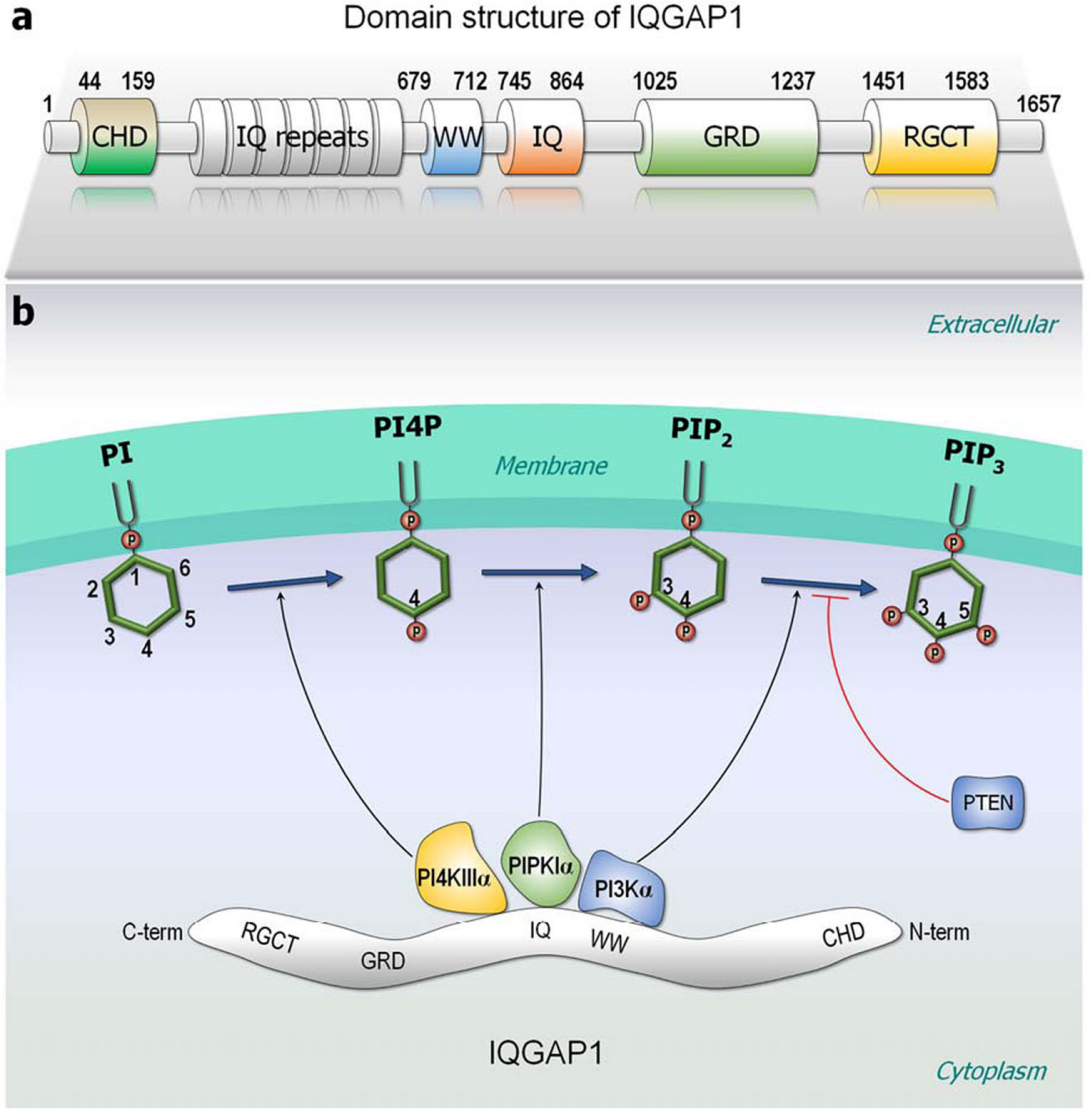
Domain structure of IQGAP1 and its scaffolding lipid kinases. **a** IQGAP1 is a large scaffolding protein and contains multi-domains, including calponin-homology domain (CHD), IQ repeats, polyproline binding region (WW), four IQ motifs (IQ), Ras GTPase-activating protein-related domain (GRD), and RasGAP C-terminus (RGCT). **b** IQGAP1 is a key regulator of phosphoinositide signaling, scaffolding various lipid kinases at the nearby plasma membrane. The lipid kinase PI4KIIIα converts PI → PI4P, PIPKIα converts PI4P → PIP_2_, and PI3Kα converts PIP_2_ → PIP_3_ at the plasma membrane. The tumor suppressor, phosphatase, and tensin homolog (PTEN) can reverse the PIP_3_ production

PIP_3_ is sequentially generated by phosphatidylinositol 4-kinase IIIα (PI4KIIIα, a.k.a. PI4KA), phosphatidylinositol 4-phosphate 5-kinase Iα (PIPKIα, a.k.a. PIP5KIα), and PI3Kα lipid kinases; all scaffolded by IQGAP1 (Fig. 8a). IQGAP1 is a 190-kDa protein involved in regulating processes such as organization of the actin cytoskeleton, transcription, and cellular adhesion to regulating the cell cycle. It can interact with over 160 proteins, including PI3K, CaM, Cdc42, and Rac (Choi and Anderson 2016; Choi et al. 2016; Hedman et al. 2015; Ozdemir et al. 2018; Smith et al. 2015) and mediates actin-binding. The first step, the conversion of phosphatidylinositol (PI) to phosphatidylinositol 4-phosphate (PI4P), the precursor of PIP_2_ and PIP_3_, is catalyzed by the PI4KIIIα complex (Fig. 8b). Truncation mutants indicated that PIPKIα interacts with the IQ domain, while the p85 subunit of PI3Kα interacts with both the WW and IQ domains of IQGAP1 (Choi et al. 2016). Scaffolding these kinases into functional proximity generates PIP_2_ and subsequently PIP_3_, which then signals to phosphoinositide-dependent protein kinase 1 (PDK1) and Akt, that are also in the complex. Blocking the interaction of IQGAP1 with PIPKIα or PI3K inhibits generation of PIP_3_ and signaling. The four IQ motifs of IQGAP1 can interact with apo- or holo- CaM (Atcheson et al. 2011; Li and Sacks 2003; White et al. 2009). IQGAP1’s WW domain interacts with the SH2 domains and may thus substitute for CaM in oncogenic Ras activation (Choi et al. 2016). IQGAP1 lacks a membrane-binding domain. It may be autoinhibited in several sites. For example, Cdc42 binding to the Ex-domain of GTPase binding domain (GRD) of IQGAP2 (GRD2) releases the Ex-domain at the C-terminal region of GRD2, relieving the autoinhibition thus facilitating IQGAP2 dimerization. Cdc42 binding promotes allosteric conformational changes in the RasGAP site, resulting in a binding site for the second Cdc42 in the RasGAP site (Ozdemir et al. 2018).

Raf (along with its mitogen-activated protein kinase kinase (MEK) substrate and the subsequent extracellular signal-regulated kinase (ERK) in the MAPK pathway) can also be scaffolded by IQGAP1; however, their major scaffolding protein is kinase suppressor of Ras (KSR). Raf family pseudokinases KSR1 and KSR2 can support the interaction of Raf isoforms with MEK. Unlike Raf, KSR lacks a Ras-binding domain; however, its dimerization with Raf stimulates Raf’s catalysis (Lavoie et al. 2018). As a peudokinase resembling Raf, KSR is also autoinhibited with the inhibition relieved by Ser406 dephosphorylation, 14-3-3 release and membrane attachment (Lavoie and Therrien 2015).

In addition to increasing the effective local concentration, enhancing, and stabilizing molecular adjacency, allosteric actions in scaffolding proteins are also expected to promote catalytic activity (Nussinov et al. 2013a), including in IQGAP1–lipid kinases interaction, and the KSR. Members of the galectin family can scaffold Ras isoforms (Rotblat et al. 2010) and have also been proposed as cancer targets (Rabinovich 2005). However, their detailed actions are unclear.

## Conclusions

Activation of Raf, PI3Kα, and NORE1A involves release of autoinhibition at the membrane; however, the distinct mechanisms differ. Activation of Raf kinase domains requires dimerization, which necessitates their spatial proximity, along with the high affinity Ras–Raf interaction which drives the shift in the population from the inactive to the active, “open” Raf state (Fig. 4). By contrast, PI3Kα kinase domain is primarily activated by the release of the autoinhibition by the high-affinity binding of the phosphorylated RTK motif, with activation assisted by active Ras (Fig. 5c). Raf is a protein kinase; PI3Kα is a lipid kinase. Raf is activated by autophosphorylation, and its substrate is MEK; by contrast, PI3Kα’s substrate, PIP_2_, is a membrane-anchored lipid, which has been synthesized by sequential catalytic actions by lipid kinases. Even though PI3Kα is a dimer, p85α is a regulatory subunit. In the cell, it mostly keeps the p110 catalytic kinase subunit in the inactive autoinhibited state. Through release of the autoinhibition of its SH2 domains, it triggers activation. The lower affinity of its RBD to Ras suggests that as long as Ras predominantly exists as dimers or nanoclusters, Raf signaling will go through (Nussinov et al. 2018b). In the cell cycle, Raf’s activation and MAPK signaling precede PI3Kα/Akt, (Nussinov et al. 2018a): MAPK works in the first phase of the G1 stage; PI3Kα/Akt in the second (Nussinov et al. 2016c; Nussinov et al. 2015b). Both pathways are required to go through the checkpoint to proceed to the Synthesis, S stage. NORE1A’s scenario resembles Raf’s; except that the release of the inhibition does not emerge from the micromolar Ras binding, but from SARAH domain heterodimerization with MST1/2, with the ultimate activation being that of the MST1/2, through its kinase domains dimerization, mediated by NORE1A (Fig. 6). If Ras expression is sufficiently high, it can form dimers (or nanoclusters). These, coupled with Raf’s high affinity, boost Raf’s activation and initiate the MAPK signaling cascade. PI3Kα activation does not require nanoclustering. However, it requires proximity to RTK (possibly accomplished by sharing membrane lipid rafts) to directly release its autoinhibition, via the c/n/SH2 domains, and shift the equilibrium.

It has been suggested that proteins regulated by autoinhibitory domains can be promising targets for allosteric drugs that stabilize the native, autoinhibited fold (Peterson and Golemis 2004). Successful examples that we could find are imatinib (Gleevec, STI-571) and wiskostatin. The similarity between the conformation of the activation loop in the imatinib-Abl structure and the substrate-binding mode suggested that imatinib bound to a native autoinhibited conformation of Abl. The GTPase binding domain (GBD) of Neural Wiskott-Aldrich syndrome protein (N-WASP) interacts with its C-terminus, autoinhibiting the activation of the Arp2/3 complex (Panchal et al. 2003). Binding of Cdc42 to the GBD relieves this autoinhibition (Abdul-Manan et al. 1999; Kim et al. 2000). Two inhibitors of N-WASP (Peterson et al. 2004), 187-1 and wiskostatin, were identified. The 187-1 inhibitor stabilizes the autoinhibitory interaction of N-WASP against activation by Cdc42. The solution structure of wiskostatin bound to the WASP (Peterson et al. 2004) suggested that wiskostatin stabilizes the autoinhibited fold of the GBD. The isolated GBD was disordered in solution. Wiskostatin induced folding of the GBD into the autoinhibited conformation, suggesting that it stabilized the native autoinhibited fold of WASP (Peterson and Golemis 2004). Nonetheless, in the case of Ras effectors discussed here, with a priori loose interaction, retaining the autoinhibition of Raf will be challenging; as is also the case for PI3Kα.
